# Ethyl 4-(*tert*-butyl­amino)-3-nitro­benzoate

**DOI:** 10.1107/S160053680802206X

**Published:** 2008-07-19

**Authors:** Siti Marina Mohd. Maidin, Aisyah Saad Abdul Rahim, Hasnah Osman, Reza Kia, Hoong-Kun Fun

**Affiliations:** a School of Pharmaceutical Sciences, Universiti Sains Malaysia, 11800 USM, Penang, Malaysia; b School of Chemical Sciences, Universiti Sains Malaysia, 11800 USM, Penang, Malaysia; c X-ray Crystallography Unit, School of Physics, Universiti Sains Malaysia, 11800 USM, Penang, Malaysia

## Abstract

In the title compound, C_13_H_18_N_2_O_4_, intra­molecular N—H⋯O, N—H⋯N and C—H⋯O (× 3) hydrogen bonds generate *S*(6) and *S*(5) ring motifs. There are two crystallographically independent mol­ecules (*A* and *B*) in the asymmetric unit. The nitro group is coplanar with the benzene ring, with O—N—C—C torsion angles of −0.33 (13) and 0.93 (14)° in mol­ecules *A* and *B*, respectively. In the crystal structure, neighbouring mol­ecules are linked together by inter­molecular C—H⋯O hydrogen bonds. In addition, the crystal structure is stabilized by π–π inter­actions with centroid–centroid distances ranging from 3.7853 (6) to 3.8625 (6) Å.

## Related literature

For literature on hydrogen-bond motifs, see: Bernstein *et al.* (1995 ▶). For values of bond lengths, see: Allen *et al.* (1987 ▶). For related literature, see, for example: Göker *et al.* (1998 ▶); Anderson (2005 ▶); Kakei *et al.* (1993 ▶).
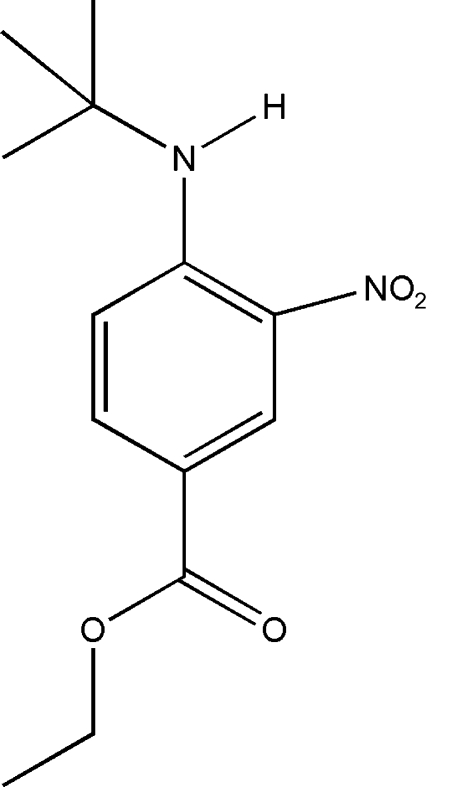


## Experimental

### 

#### Crystal data

C_13_H_18_N_2_O_4_
*M* *_r_* = 266.29
Monoclinic, 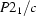

*a* = 16.0471 (5) Å
*b* = 6.6417 (2) Å
*c* = 30.0180 (9) Å
β = 121.688 (2)°
*V* = 2722.37 (14) Å^3^
*Z* = 8
Mo *K*α radiation
μ = 0.10 mm^−1^
*T* = 100.0 (1) K
0.51 × 0.43 × 0.17 mm

#### Data collection

Bruker SMART APEXII CCD area-detector diffractometer
Absorption correction: multi-scan (*SADABS*; Bruker, 2005 ▶) *T*_min_ = 0.879, *T*_max_ = 0.984
63326 measured reflections
8141 independent reflections
6368 reflections with *I* > 2σ(*I*)
*R*_int_ = 0.033

#### Refinement

*R*[*F* ^2^ > 2σ(*F* ^2^)] = 0.046
*wR*(*F* ^2^) = 0.133
*S* = 1.04
8141 reflections
351 parameters
H-atom parameters constrained
Δρ_max_ = 0.53 e Å^−3^
Δρ_min_ = −0.24 e Å^−3^

### 

Data collection: *APEX2* (Bruker, 2005 ▶); cell refinement: *APEX2*; data reduction: *SAINT* (Bruker, 2005 ▶); program(s) used to solve structure: *SHELXTL* (Sheldrick, 2008 ▶); program(s) used to refine structure: *SHELXTL*; molecular graphics: *SHELXTL*; software used to prepare material for publication: *SHELXTL* and *PLATON* (Spek, 2003 ▶).

## Supplementary Material

Crystal structure: contains datablocks global, I. DOI: 10.1107/S160053680802206X/at2594sup1.cif

Structure factors: contains datablocks I. DOI: 10.1107/S160053680802206X/at2594Isup2.hkl

Additional supplementary materials:  crystallographic information; 3D view; checkCIF report

## Figures and Tables

**Table 1:** Selected centroid–centroid distances (Å) *Cg*1 and *Cg*2 are the centroids of the C1*A*–C6*A* and C1*B*–C6*B* rings, respectively.

*Cg*1⋯*Cg*2^i^	3.7853 (6)
*Cg*1⋯*Cg*2^ii^	3.8625 (6)

Symmetry codes: (i) 
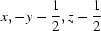
; (ii) 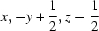.

**Table 2:** Hydrogen-bond geometry (Å, °)

*D*—H⋯*A*	*D*—H	H⋯*A*	*D*⋯*A*	*D*—H⋯*A*
N2*A*—H2*NA*⋯O4*A*	0.86	1.93	2.6299 (15)	138
N2*A*—H2*NA*⋯N1*A*	0.86	2.54	2.9361 (15)	109
N2*B*—H2*NB*⋯O4*B*	0.87	1.95	2.6355 (15)	134
N2*B*—H2*NB*⋯N1*B*	0.87	2.58	2.9419 (15)	106
C1*A*—H1*A*⋯O3*A*	0.95	2.31	2.6522 (16)	100
C1*B*—H1*B*⋯O3*B*	0.95	2.31	2.6498 (16)	100
C4*A*—H4*A*⋯O3*B*^iii^	0.95	2.50	3.4124 (17)	160
C4*B*—H4*B*⋯O3*A*^iv^	0.95	2.42	3.2566 (18)	147
C5*A*—H5*A*⋯O1*A*	0.95	2.41	2.7326 (13)	100
C11*A*—H11*A*⋯O2*B*^v^	0.98	2.55	3.4714 (17)	157
C11*B*—H11*F*⋯O2*A*^ii^	0.98	2.52	3.4446 (17)	158
C13*B*—H13*D*⋯O2*A*^vi^	0.98	2.60	3.5071 (17)	154

Symmetry codes: (ii) 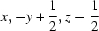; (iii) 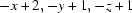; (iv) 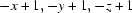; (v) 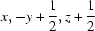; (vi) 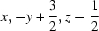.

**Fig. 1.**
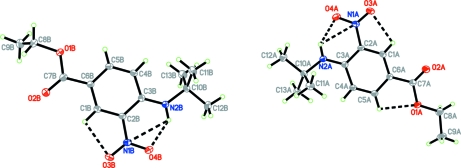
The molecular structure of the title compound, showing 50% probability displacement ellipsoids and the atomic numbering. Intramolecular hydrogen bonds are drawn as dashed lines.

**Fig. 2.**
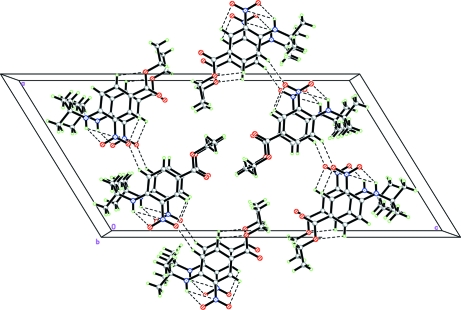
The crystal packing of (I), showing stacking arrangement viewed down the b-axis. Intramolecular and intermolecular interactions are drawn as dashed lines.

## supplementary crystallographic information

### Comment

As a part of our ongoing studies on new nitro benzoic acid derivatives, we have synthesized the title compound (I) employing a modified protocol of previous procedure (Göker *et al.*, 1998). The nitro benzoic acid intermediates are a convenient starting material for the synthesis of heterocycles targeting important biological processes, *e.g.* antifungal (Fluconazole) (Anderson, 2005) and proton pump inhibitor (Omeprazole) (Kakei *et al.*, 1993). The crystal structure of the *tert*-butylamino functionalized nitro benzoic acid (I) has been determined, and herein, we present a full report on its crystal structure.

In the title compound (I) (Fig. 1), intramolecular N—H···O (*x* 2), N—H···N (*x* 2), and C—H···O (*x* 3) hydrogen bonds (Table 2) generate *S*(6) and *S*(5) ring motifs, respectively (Bernstein *et al.*, 1995). There are two crystallographically independent molecules, A and B in the asymmetric unit of the title compound. The nitro group is coplanar with the benzene ring with torsion angle of -0.33 (13) and 0.93 (14)° in the molecule A and B, respectively. In the crystal structure neighbouring molecules are linked together by intermolecular C—H···O hydrogen bonds (Table 1). In the crystal packing (Table 2 & Fig. 2), molecules are stacked down the *b* axis, being consolidated by π–π interactions with centroid to centroid distances ranging from 3.7853 (6) – 3.8625 (6) Å.

The crystal structure is stabilized by intramolecular N—H···O (*x* 2), N—H···N (*x* 2), C—H···O (*x* 3), and intermolecular C—H···O (*x* 5) hydrogen bonds and π–π interactions.

### Experimental

Ethyl 4-fluoro-3-nitrobenzoate (200 mg, 0.93 mmol) was dissolved in dry dichloromethane (10 ml). *N*, *N*-diisopropylethylamine (DIPEA) (0.20 ml, 1.12 mmol) was added to the stirred mixture. Then, *tert*-butylamine (0.11 ml, 1.03 mmol) was added dropwise using syringe and stirred at room temperature under N_2_ overnight. After completion of the reaction, the mixture was washed with 10% NaCO_3_ (10 ml). The aqueous layer was washed with dichloromethane (3 x 15 ml). The organic layers were collected and dried over MgSO_4 _(anhydrous). The solvent was removed under reduced pressure to yield the crude product. Recrystallisation with hot hexane revealed the title compound (I) as bright yellow crystals.

### Refinement

The H-atoms attached to N2A and N2B were located from the difference Fourier map and refined as riding with the parent atom with an isotropic thermal parameter 1.2 times that of the parent atom. The rest of the hydrogen atoms were positioned geometrically [C—H = 0.95–98 Å] and refined using a riding model. A rotating-group model was used for the methyl groups. The highest peak is located 0.63 Å from C6B and the deepest hole is located 0.59 Å from N1A.

### Crystal data

**Table:**

C_13_H_18_N_2_O_4_	*F*_000_ = 1136
*M**_r_* = 266.29	*D*_x_ = 1.299 Mg m^−^^3^
Monoclinic, *P*2_1_/*c*	Mo *K*α radiation λ = 0.71073 Å
Hall symbol: -P 2ybc	Cell parameters from 9969 reflections
*a* = 16.0471 (5) Å	θ = 2.5–30.1º
*b* = 6.6417 (2) Å	µ = 0.10 mm^−^^1^
*c* = 30.0180 (9) Å	*T* = 100.0 (1) K
β = 121.688 (2)º	Plate, yellow
*V* = 2722.37 (14) Å^3^	0.51 × 0.43 × 0.17 mm
*Z* = 8	

### Data collection

**Table:**

Bruker SMART APEXII CCD area-detector diffractometer	8141 independent reflections
Radiation source: fine-focus sealed tube	6368 reflections with *I* > 2σ(*I*)
Monochromator: graphite	*R*_int_ = 0.033
*T* = 100.0(1) K	θ_max_ = 30.3º
φ and ω scans	θ_min_ = 1.5º
Absorption correction: multi-scan(SADABS; Bruker, 2005)	*h* = −21→22
*T*_min_ = 0.879, *T*_max_ = 0.984	*k* = −9→9
63326 measured reflections	*l* = −41→42

### Refinement

**Table:**

Refinement on *F*^2^	Secondary atom site location: difference Fourier map
Least-squares matrix: full	Hydrogen site location: inferred from neighbouring sites
*R*[*F*^2^ > 2σ(*F*^2^)] = 0.047	H-atom parameters constrained
*wR*(*F*^2^) = 0.133	*w* = 1/[σ^2^(*F*_o_^2^) + (0.0652*P*)^2^ + 0.787*P*] where *P* = (*F*_o_^2^ + 2*F*_c_^2^)/3
*S* = 1.04	(Δ/σ)_max_ = 0.001
8141 reflections	Δρ_max_ = 0.53 e Å^−^^3^
351 parameters	Δρ_min_ = −0.24 e Å^−^^3^
Primary atom site location: structure-invariant direct methods	Extinction correction: none

### Special details

**Table:**

Experimental. The low-temperature data was collected with the Oxford Cyrosystem Cobra low-temperature attachment.
Geometry. All e.s.d.'s (except the e.s.d. in the dihedral angle between two l.s. planes) are estimated using the full covariance matrix. The cell e.s.d.'s are taken into account individually in the estimation of e.s.d.'s in distances, angles and torsion angles; correlations between e.s.d.'s in cell parameters are only used when they are defined by crystal symmetry. An approximate (isotropic) treatment of cell e.s.d.'s is used for estimating e.s.d.'s involving l.s. planes.
Refinement. Refinement of *F*^2^ against ALL reflections. The weighted *R*-factor *wR* and goodness of fit *S* are based on *F*^2^, conventional *R*-factors *R* are based on *F*, with *F* set to zero for negative *F*^2^. The threshold expression of *F*^2^ > σ(*F*^2^) is used only for calculating *R*-factors(gt) *etc*. and is not relevant to the choice of reflections for refinement. *R*-factors based on *F*^2^ are statistically about twice as large as those based on *F*, and *R*- factors based on ALL data will be even larger.

### Fractional atomic coordinates and isotropic or equivalent isotropic displacement parameters (Å^2^)

**Table:**

	*x*	*y*	*z*	*U*_iso_*/*U*_eq_	
O1A	1.01188 (6)	0.50707 (13)	0.90700 (3)	0.02027 (18)	
O2A	0.87073 (6)	0.53054 (14)	0.90534 (3)	0.02371 (19)	
O3A	0.57249 (6)	0.54152 (13)	0.73478 (3)	0.02243 (18)	
O4A	0.56803 (6)	0.54680 (13)	0.66152 (3)	0.02250 (18)	
N1A	0.61441 (6)	0.54228 (13)	0.71013 (4)	0.01589 (18)	
N2A	0.73120 (6)	0.54415 (13)	0.66080 (3)	0.01583 (18)	
H2NA	0.6682	0.5497	0.6444	0.019*	
C1A	0.76647 (7)	0.53258 (15)	0.79329 (4)	0.0143 (2)	
H1A	0.7281	0.5335	0.8089	0.017*	
C2A	0.72036 (7)	0.53705 (15)	0.73898 (4)	0.01349 (19)	
C3A	0.77441 (7)	0.53716 (14)	0.71324 (4)	0.01347 (19)	
C4A	0.87825 (7)	0.52932 (15)	0.74731 (4)	0.0151 (2)	
H4A	0.9180	0.5271	0.7325	0.018*	
C5A	0.92253 (7)	0.52490 (15)	0.80055 (4)	0.0149 (2)	
H5A	0.9920	0.5204	0.8217	0.018*	
C6A	0.86747 (7)	0.52682 (15)	0.82473 (4)	0.0142 (2)	
C7A	0.91408 (8)	0.52224 (16)	0.88226 (4)	0.0159 (2)	
C8A	1.06291 (8)	0.5008 (2)	0.96363 (4)	0.0252 (3)	
H8A	1.0412	0.3828	0.9751	0.030*	
H8B	1.0489	0.6242	0.9771	0.030*	
C9A	1.17025 (9)	0.4861 (2)	0.98412 (5)	0.0347 (3)	
H9A	1.2069	0.4877	1.0225	0.052*	
H9B	1.1904	0.6006	0.9713	0.052*	
H9C	1.1837	0.3603	0.9720	0.052*	
C10A	0.77667 (8)	0.54745 (16)	0.62878 (4)	0.0163 (2)	
C11A	0.83246 (8)	0.35165 (17)	0.63510 (4)	0.0203 (2)	
H11A	0.7877	0.2370	0.6254	0.030*	
H11B	0.8861	0.3373	0.6716	0.030*	
H11C	0.8593	0.3555	0.6124	0.030*	
C12A	0.68959 (9)	0.56150 (19)	0.57218 (4)	0.0233 (2)	
H12A	0.6458	0.4465	0.5647	0.035*	
H12B	0.7136	0.5600	0.5481	0.035*	
H12C	0.6537	0.6869	0.5676	0.035*	
C13A	0.84047 (8)	0.73502 (17)	0.64034 (4)	0.0195 (2)	
H13A	0.8927	0.7359	0.6774	0.029*	
H13B	0.8000	0.8560	0.6324	0.029*	
H13C	0.8695	0.7331	0.6186	0.029*	
O1B	0.49200 (5)	0.43211 (13)	0.10402 (3)	0.02139 (18)	
O2B	0.63293 (6)	0.45058 (13)	0.10484 (3)	0.02286 (18)	
O3B	0.93204 (6)	0.47400 (13)	0.27341 (3)	0.02111 (18)	
O4B	0.93898 (6)	0.47557 (13)	0.34743 (3)	0.02151 (18)	
N1B	0.89147 (6)	0.47252 (13)	0.29872 (4)	0.01529 (18)	
N2B	0.77682 (6)	0.47199 (14)	0.34950 (3)	0.01565 (18)	
H2NB	0.8407	0.4767	0.3671	0.019*	
C1B	0.73817 (7)	0.46115 (15)	0.21633 (4)	0.01401 (19)	
H1B	0.7757	0.4608	0.2002	0.017*	
C2B	0.78566 (7)	0.46722 (15)	0.27069 (4)	0.01293 (19)	
C3B	0.73273 (7)	0.46737 (15)	0.29702 (4)	0.01340 (19)	
C4B	0.62889 (8)	0.46322 (16)	0.26366 (4)	0.0157 (2)	
H4B	0.5900	0.4645	0.2790	0.019*	
C5B	0.58317 (7)	0.45740 (15)	0.21029 (4)	0.0154 (2)	
H5B	0.5137	0.4545	0.1895	0.019*	
C6B	0.63721 (7)	0.45564 (15)	0.18553 (4)	0.01401 (19)	
C7B	0.58976 (8)	0.44669 (16)	0.12800 (4)	0.0168 (2)	
C8B	0.43816 (8)	0.4179 (2)	0.04737 (4)	0.0260 (3)	
H8C	0.3751	0.3484	0.0350	0.031*	
H8D	0.4762	0.3370	0.0365	0.031*	
C9B	0.41883 (9)	0.6226 (2)	0.02271 (5)	0.0302 (3)	
H9D	0.3804	0.6089	−0.0155	0.045*	
H9E	0.4812	0.6888	0.0335	0.045*	
H9F	0.3822	0.7038	0.0340	0.045*	
C10B	0.73206 (8)	0.46992 (16)	0.38189 (4)	0.0172 (2)	
C11B	0.67381 (8)	0.27595 (17)	0.37340 (4)	0.0217 (2)	
H11D	0.6189	0.2698	0.3370	0.032*	
H11E	0.6487	0.2747	0.3969	0.032*	
H11F	0.7166	0.1593	0.3809	0.032*	
C12B	0.81999 (9)	0.47351 (19)	0.43837 (4)	0.0246 (2)	
H12D	0.8582	0.5964	0.4441	0.037*	
H12E	0.8613	0.3555	0.4445	0.037*	
H12F	0.7966	0.4710	0.4627	0.037*	
C13B	0.66997 (8)	0.65807 (18)	0.37269 (5)	0.0222 (2)	
H13D	0.7108	0.7784	0.3806	0.033*	
H13E	0.6433	0.6541	0.3955	0.033*	
H13F	0.6160	0.6623	0.3360	0.033*	

### Atomic displacement parameters (Å^2^)

**Table:**

	*U*^11^	*U*^22^	*U*^33^	*U*^12^	*U*^13^	*U*^23^
O1A	0.0132 (3)	0.0327 (5)	0.0134 (4)	0.0004 (3)	0.0059 (3)	−0.0014 (3)
O2A	0.0181 (4)	0.0365 (5)	0.0190 (4)	0.0000 (3)	0.0114 (3)	−0.0013 (3)
O3A	0.0154 (4)	0.0298 (5)	0.0260 (4)	0.0006 (3)	0.0135 (3)	0.0003 (3)
O4A	0.0136 (4)	0.0309 (5)	0.0186 (4)	0.0002 (3)	0.0055 (3)	−0.0002 (3)
N1A	0.0126 (4)	0.0142 (4)	0.0205 (4)	−0.0004 (3)	0.0084 (3)	−0.0004 (3)
N2A	0.0120 (4)	0.0192 (4)	0.0151 (4)	0.0004 (3)	0.0062 (3)	0.0003 (3)
C1A	0.0149 (5)	0.0111 (4)	0.0189 (5)	−0.0006 (3)	0.0104 (4)	−0.0004 (4)
C2A	0.0102 (4)	0.0119 (5)	0.0175 (5)	0.0002 (3)	0.0068 (4)	0.0002 (4)
C3A	0.0135 (4)	0.0099 (4)	0.0168 (5)	−0.0006 (3)	0.0078 (4)	−0.0003 (3)
C4A	0.0127 (4)	0.0155 (5)	0.0182 (5)	0.0003 (4)	0.0088 (4)	0.0005 (4)
C5A	0.0124 (4)	0.0145 (5)	0.0175 (5)	0.0003 (4)	0.0076 (4)	0.0006 (4)
C6A	0.0147 (5)	0.0131 (5)	0.0148 (5)	−0.0004 (4)	0.0077 (4)	−0.0005 (4)
C7A	0.0140 (5)	0.0160 (5)	0.0170 (5)	−0.0009 (4)	0.0075 (4)	−0.0013 (4)
C8A	0.0188 (5)	0.0421 (7)	0.0122 (5)	0.0004 (5)	0.0065 (4)	−0.0021 (5)
C9A	0.0184 (6)	0.0630 (10)	0.0180 (6)	0.0030 (6)	0.0063 (5)	−0.0027 (6)
C10A	0.0161 (5)	0.0183 (5)	0.0154 (5)	−0.0005 (4)	0.0088 (4)	0.0001 (4)
C11A	0.0208 (5)	0.0181 (5)	0.0240 (5)	−0.0002 (4)	0.0131 (4)	−0.0028 (4)
C12A	0.0212 (5)	0.0303 (6)	0.0157 (5)	−0.0009 (5)	0.0078 (4)	−0.0005 (4)
C13A	0.0205 (5)	0.0190 (5)	0.0203 (5)	−0.0011 (4)	0.0116 (4)	0.0018 (4)
O1B	0.0133 (4)	0.0343 (5)	0.0138 (4)	−0.0022 (3)	0.0053 (3)	0.0013 (3)
O2B	0.0181 (4)	0.0339 (5)	0.0178 (4)	0.0012 (3)	0.0102 (3)	−0.0004 (3)
O3B	0.0150 (4)	0.0280 (4)	0.0238 (4)	0.0003 (3)	0.0127 (3)	0.0009 (3)
O4B	0.0136 (4)	0.0302 (5)	0.0164 (4)	0.0002 (3)	0.0049 (3)	0.0004 (3)
N1B	0.0135 (4)	0.0134 (4)	0.0186 (4)	0.0001 (3)	0.0081 (3)	0.0002 (3)
N2B	0.0129 (4)	0.0195 (4)	0.0143 (4)	0.0001 (3)	0.0070 (3)	0.0001 (3)
C1B	0.0144 (4)	0.0125 (5)	0.0168 (5)	0.0004 (3)	0.0093 (4)	0.0006 (4)
C2B	0.0108 (4)	0.0113 (4)	0.0166 (5)	0.0003 (3)	0.0072 (4)	0.0007 (3)
C3B	0.0147 (5)	0.0101 (4)	0.0151 (5)	0.0004 (3)	0.0077 (4)	0.0010 (3)
C4B	0.0137 (4)	0.0173 (5)	0.0185 (5)	0.0004 (4)	0.0100 (4)	0.0005 (4)
C5B	0.0121 (4)	0.0151 (5)	0.0185 (5)	0.0000 (4)	0.0076 (4)	0.0007 (4)
C6B	0.0135 (4)	0.0133 (5)	0.0148 (5)	0.0000 (3)	0.0071 (4)	0.0009 (3)
C7B	0.0152 (5)	0.0170 (5)	0.0163 (5)	0.0001 (4)	0.0070 (4)	0.0007 (4)
C8B	0.0176 (5)	0.0410 (7)	0.0148 (5)	−0.0037 (5)	0.0055 (4)	−0.0017 (5)
C9B	0.0236 (6)	0.0469 (8)	0.0197 (6)	0.0022 (5)	0.0111 (5)	0.0074 (5)
C10B	0.0194 (5)	0.0187 (5)	0.0158 (5)	0.0009 (4)	0.0107 (4)	0.0007 (4)
C11B	0.0255 (6)	0.0209 (5)	0.0226 (5)	−0.0013 (4)	0.0154 (5)	0.0024 (4)
C12B	0.0265 (6)	0.0306 (6)	0.0152 (5)	0.0009 (5)	0.0099 (5)	0.0008 (4)
C13B	0.0242 (5)	0.0205 (5)	0.0251 (6)	0.0019 (4)	0.0151 (5)	−0.0022 (4)

### Geometric parameters (Å, °)

**Table:**

O1A—C7A	1.3418 (13)	O1B—C7B	1.3424 (13)
O1A—C8A	1.4489 (13)	O1B—C8B	1.4503 (13)
O2A—C7A	1.2136 (13)	O2B—C7B	1.2125 (13)
O3A—N1A	1.2340 (12)	O3B—N1B	1.2332 (12)
O4A—N1A	1.2424 (12)	O4B—N1B	1.2443 (12)
N1A—C2A	1.4474 (13)	N1B—C2B	1.4462 (13)
N2A—C3A	1.3470 (13)	N2B—C3B	1.3463 (13)
N2A—C10A	1.4810 (13)	N2B—C10B	1.4816 (13)
N2A—H2NA	0.8617	N2B—H2NB	0.8733
C1A—C6A	1.3827 (14)	C1B—C6B	1.3809 (14)
C1A—C2A	1.3925 (14)	C1B—C2B	1.3924 (14)
C1A—H1A	0.9500	C1B—H1B	0.9500
C2A—C3A	1.4328 (14)	C2B—C3B	1.4321 (14)
C3A—C4A	1.4265 (14)	C3B—C4B	1.4238 (14)
C4A—C5A	1.3663 (14)	C4B—C5B	1.3683 (14)
C4A—H4A	0.9500	C4B—H4B	0.9500
C5A—C6A	1.4076 (14)	C5B—C6B	1.4076 (14)
C5A—H5A	0.9500	C5B—H5B	0.9500
C6A—C7A	1.4784 (14)	C6B—C7B	1.4778 (14)
C8A—C9A	1.4973 (17)	C8B—C9B	1.5003 (19)
C8A—H8A	0.9900	C8B—H8C	0.9900
C8A—H8B	0.9900	C8B—H8D	0.9900
C9A—H9A	0.9800	C9B—H9D	0.9800
C9A—H9B	0.9800	C9B—H9E	0.9800
C9A—H9C	0.9800	C9B—H9F	0.9800
C10A—C13A	1.5320 (15)	C10B—C13B	1.5302 (15)
C10A—C11A	1.5331 (15)	C10B—C11B	1.5323 (15)
C10A—C12A	1.5343 (15)	C10B—C12B	1.5338 (15)
C11A—H11A	0.9800	C11B—H11D	0.9800
C11A—H11B	0.9800	C11B—H11E	0.9800
C11A—H11C	0.9800	C11B—H11F	0.9800
C12A—H12A	0.9800	C12B—H12D	0.9800
C12A—H12B	0.9800	C12B—H12E	0.9800
C12A—H12C	0.9800	C12B—H12F	0.9800
C13A—H13A	0.9800	C13B—H13D	0.9800
C13A—H13B	0.9800	C13B—H13E	0.9800
C13A—H13C	0.9800	C13B—H13F	0.9800
			
Cg1···Cg2^i^	3.7853 (6)	Cg1···Cg2^ii^	3.8625 (6)
			
C7A—O1A—C8A	115.30 (9)	C7B—O1B—C8B	116.24 (9)
O3A—N1A—O4A	121.71 (9)	O3B—N1B—O4B	121.87 (9)
O3A—N1A—C2A	118.70 (9)	O3B—N1B—C2B	118.70 (9)
O4A—N1A—C2A	119.59 (9)	O4B—N1B—C2B	119.43 (9)
C3A—N2A—C10A	129.22 (9)	C3B—N2B—C10B	129.04 (9)
C3A—N2A—H2NA	113.4	C3B—N2B—H2NB	115.9
C10A—N2A—H2NA	117.3	C10B—N2B—H2NB	115.0
C6A—C1A—C2A	120.74 (9)	C6B—C1B—C2B	120.85 (9)
C6A—C1A—H1A	119.6	C6B—C1B—H1B	119.6
C2A—C1A—H1A	119.6	C2B—C1B—H1B	119.6
C1A—C2A—C3A	122.10 (9)	C1B—C2B—C3B	121.91 (9)
C1A—C2A—N1A	115.83 (9)	C1B—C2B—N1B	115.79 (9)
C3A—C2A—N1A	122.07 (9)	C3B—C2B—N1B	122.30 (9)
N2A—C3A—C4A	121.97 (9)	N2B—C3B—C4B	121.65 (9)
N2A—C3A—C2A	122.98 (9)	N2B—C3B—C2B	123.11 (9)
C4A—C3A—C2A	115.05 (9)	C4B—C3B—C2B	115.23 (9)
C5A—C4A—C3A	122.23 (10)	C5B—C4B—C3B	122.25 (9)
C5A—C4A—H4A	118.9	C5B—C4B—H4B	118.9
C3A—C4A—H4A	118.9	C3B—C4B—H4B	118.9
C4A—C5A—C6A	121.42 (9)	C4B—C5B—C6B	121.22 (9)
C4A—C5A—H5A	119.3	C4B—C5B—H5B	119.4
C6A—C5A—H5A	119.3	C6B—C5B—H5B	119.4
C1A—C6A—C5A	118.45 (9)	C1B—C6B—C5B	118.54 (9)
C1A—C6A—C7A	119.36 (9)	C1B—C6B—C7B	119.12 (9)
C5A—C6A—C7A	122.19 (9)	C5B—C6B—C7B	122.34 (9)
O2A—C7A—O1A	122.82 (10)	O2B—C7B—O1B	123.56 (10)
O2A—C7A—C6A	125.16 (10)	O2B—C7B—C6B	124.77 (10)
O1A—C7A—C6A	112.03 (9)	O1B—C7B—C6B	111.67 (9)
O1A—C8A—C9A	107.65 (9)	O1B—C8B—C9B	111.18 (11)
O1A—C8A—H8A	110.2	O1B—C8B—H8C	109.4
C9A—C8A—H8A	110.2	C9B—C8B—H8C	109.4
O1A—C8A—H8B	110.2	O1B—C8B—H8D	109.4
C9A—C8A—H8B	110.2	C9B—C8B—H8D	109.4
H8A—C8A—H8B	108.5	H8C—C8B—H8D	108.0
C8A—C9A—H9A	109.5	C8B—C9B—H9D	109.5
C8A—C9A—H9B	109.5	C8B—C9B—H9E	109.5
H9A—C9A—H9B	109.5	H9D—C9B—H9E	109.5
C8A—C9A—H9C	109.5	C8B—C9B—H9F	109.5
H9A—C9A—H9C	109.5	H9D—C9B—H9F	109.5
H9B—C9A—H9C	109.5	H9E—C9B—H9F	109.5
N2A—C10A—C13A	111.31 (9)	N2B—C10B—C13B	111.59 (9)
N2A—C10A—C11A	111.21 (9)	N2B—C10B—C11B	111.24 (9)
C13A—C10A—C11A	112.61 (9)	C13B—C10B—C11B	111.97 (9)
N2A—C10A—C12A	104.30 (8)	N2B—C10B—C12B	104.10 (9)
C13A—C10A—C12A	108.24 (9)	C13B—C10B—C12B	108.70 (9)
C11A—C10A—C12A	108.76 (9)	C11B—C10B—C12B	108.88 (9)
C10A—C11A—H11A	109.5	C10B—C11B—H11D	109.5
C10A—C11A—H11B	109.5	C10B—C11B—H11E	109.5
H11A—C11A—H11B	109.5	H11D—C11B—H11E	109.5
C10A—C11A—H11C	109.5	C10B—C11B—H11F	109.5
H11A—C11A—H11C	109.5	H11D—C11B—H11F	109.5
H11B—C11A—H11C	109.5	H11E—C11B—H11F	109.5
C10A—C12A—H12A	109.5	C10B—C12B—H12D	109.5
C10A—C12A—H12B	109.5	C10B—C12B—H12E	109.5
H12A—C12A—H12B	109.5	H12D—C12B—H12E	109.5
C10A—C12A—H12C	109.5	C10B—C12B—H12F	109.5
H12A—C12A—H12C	109.5	H12D—C12B—H12F	109.5
H12B—C12A—H12C	109.5	H12E—C12B—H12F	109.5
C10A—C13A—H13A	109.5	C10B—C13B—H13D	109.5
C10A—C13A—H13B	109.5	C10B—C13B—H13E	109.5
H13A—C13A—H13B	109.5	H13D—C13B—H13E	109.5
C10A—C13A—H13C	109.5	C10B—C13B—H13F	109.5
H13A—C13A—H13C	109.5	H13D—C13B—H13F	109.5
H13B—C13A—H13C	109.5	H13E—C13B—H13F	109.5

Symmetry codes: (i) *x*, −*y*−1/2, *z*−1/2; (ii) *x*, −*y*+1/2, *z*−1/2.

### Hydrogen-bond geometry (Å, °)

**Table:**

*D*—H···*A*	*D*—H	H···*A*	*D*···*A*	*D*—H···*A*
N2A—H2NA···O4A	0.86	1.93	2.6299 (15)	138
N2A—H2NA···N1A	0.86	2.54	2.9361 (15)	109
N2B—H2NB···O4B	0.87	1.95	2.6355 (15)	134
N2B—H2NB···N1B	0.87	2.58	2.9419 (15)	106
C1A—H1A···O3A	0.95	2.31	2.6522 (16)	100
C1B—H1B···O3B	0.95	2.31	2.6498 (16)	100
C4A—H4A···O3B^iii^	0.95	2.50	3.4124 (17)	160
C4B—H4B···O3A^iv^	0.95	2.42	3.2566 (18)	147
C5A—H5A···O1A	0.95	2.41	2.7326 (13)	100
C11A—H11A···O2B^v^	0.98	2.55	3.4714 (17)	157
C11B—H11F···O2A^ii^	0.98	2.52	3.4446 (17)	158
C13B—H13D···O2A^vi^	0.98	2.60	3.5071 (17)	154

Symmetry codes: (iii) −*x*+2, −*y*+1, −*z*+1; (iv) −*x*+1, −*y*+1, −*z*+1; (v) *x*, −*y*+1/2, *z*+1/2; (ii) *x*, −*y*+1/2, *z*−1/2; (vi) *x*, −*y*+3/2, *z*−1/2.
